# Characterization of feline-originated probiotics *Lactobacillus rhamnosus* CACC612 and *Bifidobacterium animalis* subsp. *lactis* CACC789 and evaluation of their host response

**DOI:** 10.1186/s12917-024-03975-3

**Published:** 2024-04-01

**Authors:** Hyun-Jun Jang, Jung-Ae Kim, Yangseon Kim

**Affiliations:** Department of Research and Development, Center for Industrialization of Agricultural and Livestock Microorganisms, Jeongeup-si, South Korea

**Keywords:** Probiotic characterization, Animal health, Feline, Immune

## Abstract

**Background:**

Probiotics are beneficial for animal health and new potential probiotics need to be characterized for their prospective use in improving animal health. In this study, 32 bacterial strains were isolated from a Norwegian forest cat (castrated, 12 years old) and a Persian cat (castrated, 10 years old), which were privately owned and had indoor access.

**Results:**

*Lactobacillus rhamnosus* CACC612 (CACC612) and *Bifidobacterium animalis* subsp. *lactis* CACC789 (CACC789) were selected as potential probiotics; characterization of the two strains showed equivalent acid tolerance, similar cell adhesion rates on the HT-29 monolayer cell line, and superior bile tolerance compared to *Lactobacillus rhamnosus* GG (LGG). Subsequently, they exhibited inhibitory effects against a broad spectrum of pathogenic bacteria, including *E. coli* (KCTC 2617), *Salmonella* Derby (NCCP 12,238), *Salmonella* Enteritidis (NCCP 14,546), *Salmonella* Typhimurium (NCCP 10,328), *Clostridium difficile* JCM 1296T. From evaluating host effects, the viability of the feline macrophage cell line (Fcwf-4) increased with the treatment of CACC612 or CACC789 (*P* < 0.05). The induced expression of immune-related genes such as IFN-γ, IL1β, IL2, IL4, and TNF-α by immune stimulation was significantly attenuated by the treatment of CACC612 or CACC789 (*P* < 0.05). When 52 clinical factors of sera from 21 healthy cats were analyzed using partial least squares discriminant analysis (PLS-DA), the animals were obviously clustered before and after feeding with CACC612 or CACC789. In addition, hemoglobin and mean corpuscular hemoglobin concentration (MCHC) significantly increased after CACC612 feeding (*P* < 0.05).

**Conclusions:**

In this study, feline-originated probiotics were newly characterized and their potentially probiotic effects were evaluated. These results contribute to our understanding of the functional effects of feline-derived probiotics and support their industrial applications.

## Background

The World Health Organization (WHO) defined probiotics as “live microorganisms that, when administered in adequate amounts, confer a health benefit on the host” [1]; this definition has generally been accepted and adopted in related research and industrial fields [2–4]. Many studies on probiotics have supported their potentially beneficial effects, such as improving human and animal health, modulating the intestinal microbiome, and replacing antibiotics [5]. According to this definition, probiotics are restricted to live microbes, and their number in a probiotic product is related to their effectiveness. In this regard, the minimum number of live probiotic microorganisms was suggested at least 10^9^ colony-forming units (CFUs) per day (Italian Ministry of Health) or per serving (Health Canada) [6]. *Enterococcus* spp., *Lactobacillus* spp., and *Bifidobacterium* spp., which produce lactic acid as an end product, are the most common probiotics used in animals [7]. Generally, lactic acid-producing bacteria are gram-positive anaerobes, facultative anaerobes, and non-spore-forming [8]; they can produce other substances, such as hydrogen peroxide and bacteriocins, which affect the host microbiota [9].

Relating to companion animals such as dogs and cats, the Association of American Feed Control Officials (AAFCO) announced that 41 non-toxigenic bacterial species are deemed safe for use in companion animals [10]. Among them, *Lactococcus* and *Lactobacillus* genera are mostly given the GRAS status while some other genera contain some opportunistic pathogens [11, 12]; *Bacillus* spp., *Lactobacillus* spp., *Bifidobacterium* spp., and *Enterococcus faecium* have been studied as potential probiotics for companion animals. These probiotics have been reported to have benefits for the host, such as modulation of the immune system, assistance in stress maintenance, protection from infections caused by enteropathogens, increased growth and development, and control of allergic disorders and obesity [13–21]. However, data from animal clinical trials often arouse arguments regarding the number of subjects, period, dosage, and strains used, making comparisons among studies complex [22].

Although it remains unclear, some scientists have contended that commensal microorganisms may exert host-specific effects; ideally, canine or feline probiotics derived from the gastrointestinal tract (GIT) of the animal would be effective in controlling host-specific infection in their intestines [23]. In addition, cats are obligate carnivores and require feeding with high protein content, low/moderate fat content, and a minimal amount of carbohydrates with different microbiome communities and nutrient metabolism than dogs [24]. Several studies have focused on isolating, testing, and characterizing feline-specific probiotics [14, 25].

In this study, we isolated feline-specific probiotics, including *Lactobacillus rhamnosus* CACC612 and *Bifidobacterium animalis* subsp. *lactis* CACC789, and confirmed their probiotic characteristics; they showed superior efficiency in in vitro and in vivo tests. Therefore, our data contribute to understanding the potential benefits of host-specific probiotics in cats.

## Results

### Identification of cat-originated probiotics

The 32 bacterial strains were isolated from the feces of two cats and identified using 16 S rRNA gene sequencing. From the identified strains, *Lactobacillus rhamnosus* CACC612 (CACC612, GeneBank: MZ323890.1) and *Bifidobacterium animalis* subsp. *lactis* CACC789 (CACC789, GeneBank: MZ323908.1), which are acceptable by “Regulations Concerning Recognition of Functional Ingredients and Standards and Specifications for Health Functional Foods, South Korea” were further analyzed as probiotics. As a reference strain, *Lactobacillus rhamnosus* Gorbach–Goldin ATCC53013 (LGG) was obtained from Korean Collection for Type Cultures [26] (Table 1).

**Table 1:** Lists of primers used to perform qRT-PCR

Target gene	NCBI ID	PCR product size (bp)	Sequence (5’ → 3’)	Cytokine categories [reference]
IFN-γ	NM_001009873.1	147	F-ATGTAGCAGATGGTGGGTCG	Pro-inflammatory [61]
			R-TCCTTTGAATGCGCTGGTCA	
IL1B	NM_001077414.1	161	F-AAGACGGGAAACCCACCCTA	Pro-inflammatory [62]
			R-TGCTTGAGAGGTGCTGATGT	
IL2	XM_023252215.1	270	F-AGAGCTTTCTATCAGCCTCTCT	Pro-inflammatory [63]
			R-GGCCTTCTTGGGCACGTAAA	
IL4	NM_001043339.1	133	F-GAGAAACGACTCGTGCATGG	Anti-inflammatory [62]
			R-GGTGGAGCAGTTGTGATGTG	
IL8 (CXCL8)	NM_001009281.1	172	F-GACCCCAAGCAAAAGTGGGT	Pro-inflammatory [64]
			R-ACTGCATGAAGTGCTGAAGTG	
IL10	NM_001009209.1	156	F-TCAAACCAAGGACGAGCTGC	Anti-inflammatory [62]
			R-TGTTTGATGTCTGGGTCCTCG	
IL12A	NM_001009833.1	117	F-CACACCAAGCCCAGGAATGT	Pro-inflammatory [65]
			R-TCGGAAGTGCAGGGGTAAAA	
IL12B	NM_001077413.1	185	F-TGTCAAAAGCAGCAGAGGCT	Pro-inflammatory [65]
			R-GAATAGCGTCCACCACGACT	
TNF-α	NM_001009835.1	81	F-CCCACATGGCCTGCAACTAA	Pro-inflammatory [62]
			R-GCTACTGGCTTGTCACTCGG	
GAPDH	NM_001009307	101 (genomic 173)	F-AGTATGATTCCACCCACGGCA	Not applicable
			R-GATCTCGCTCCTGGAAGATGGT	

### Acid and bile tolerance and adhesion to intestinal cell lines

Acid and bile tolerance was tested at pH 2.5 and 0.3% and 1% bile salts. CACC612 and CACC789 showed higher or equivalent survivability (CACC612, 97.9%, CACC789, 86.35%, and LGG, 44.8% at 0.3% bile salt and CACC612, 98.8%, CACC789, 84.16%, and LGG, 24.4% at 1% bile salt) at 0.3% and 1% bile salts-treated conditions, but lower survivability (CACC612, 75.9%, CACC789, 82.92%, and LGG, 98.8%) at pH 2.5 compared to LGG (*P* < 0.05) (Table 2). In addition, an assessment of the ability to adhere to the intestinal lining using the human colonic carcinoma cell line HT-29 revealed that CACC612 and CACC789 exhibited activity equivalent to that of LGG (*P* < 0.05) (Table 3). Therefore, these results suggest that the bacterial strains were tolerant to the bile salt environments and could equivalently attach to the intestinal lining relative to the reference probiotic strain; however, they were susceptible to acidic conditions.

**Table 2:** Acid and bile tolerance of feline-originated probiotics

	Condition		CACC612	CACC789	LGG
Acid tolerance	pH 2.5	0 h	7.52 ± 0.02	7.58 ± 0.26	7.74 ± 0.15
		2 h	4.40 ± 0.11	6.29 ± 0.06	7.64 ± 0.13
		Survival rate (%)	75.9	82.92	98.8
Bile tolerance	0.3% oxgall	0 h	7.52 ± 0.02	7.58 ± 0.26	7.74 ± 0.15
		2 h	7.36 ± 0.03	6.55 ± 0.06	3.47 ± 0.24
		Survival rate (%)	97.9	86.35	44.8
	1.0% oxgall	0 h	7.52 ± 0.02	7.58 ± 0.26	7.74 ± 0.15
		2 h	7.43 ± 0.02	6.38 ± 0.2	1.88 ± 0.03
		Survival rate (%)	98.8	84.16	24.4

Unit = Log_10_CFU/ml; Survivability (%) = treatment unit/control unit × 100; LGG (reference strain), *Lactobacillus rhamnosus GG* (ATCC53103)

**Table 3:** Cell adhesion activity of feline-originated probiotics on the intestinal cell line (HT-29)

Strain	0 h	After 2 h	Adherence (%)
CACC612	8.75 ± 0.03	6.86 ± 0.05	78.40
CACC789	8.06 ± 0.12	6.33 ± 0.25	78.43
LGG	7.63 ± 0.23	6.25 ± 0.11	81.93

Unit = Log_10_CFU/ml; Adhesion ability (%) = 2 h unit/0 hr unit ｘ 100; Reference strain, *Lactobacillus rhamnosus GG* (ATCC53103)

### Antibacterial activity and antibiotic sensitivity

The antibacterial activity test against various pathogenic bacteria revealed that CACC612 exhibited antibacterial activity against all tested pathogenic bacteria, including *Escherichia coli* (K99 KCTC 2617), *Salmonella* Derby (NCCP 12,238), *Salmonella* Enteritidis (NCCP 14,546), *Salmonella* Typhimurium (NCCP 10,438), and *Clostridium difficile* (JCM1296). In addition, CACC789 showed antibacterial activity against *Salmonella* Enteritidis (NCCP 14,546) and *Salmonella* Typhimurium (NCCP 10,438) (Table 4). Furthermore, based on the assessment of antibiotic sensitivity according to the European Food Safety Authority (EFSA) [27], CACC612 fulfilled the safe minimum inhibitory concentration (MIC) for all tested antibiotics, excluding gentamicin and kanamycin, and CACC789 fulfilled the safe MIC for antibiotics excluding gentamicin (Table 5). Therefore, we proposed that these bacterial strains have a wide range of pathogen-inhibitory effects and are less susceptible to concerns regarding antibiotic resistance.

**Table 4:** Antibacterial activity of feline-originated probiotics

Strain	E. coli (KCTC 2617)	Salmonella Derby (NCCP 12,238)	Salmonella Enteritidis (NCCP 14,546)	Salmonella Typhimurium (NCCP 10,328)	Clostridium difficile (JCM 1296T)
CACC612	++	+	++	+	+
CACC789	-	-	+	++	-

The inhibition zone (mm) around the paper disc containing the microbial cell-free supernatant was classified as ++, > 12 ∽ 14 mm; +, > 11 mm; -, no inhibition zone

**Table 5:** Antibiotic resistance of feline-originated probiotics

Strain	Minimal inhibition concentration (MIC, µg/mL)						
	Ampicillin	Vancomycin	Gentamicin	Kanamycin	Erythromycin	Clindamycin	Tetracycline
CACC612	2	> 256^R^	48	> 256^R^	1	0.094	8
CACC789	0.064	0.75	192	> 256^R^	0.047	< 0.016^S^	8
EFSA guideline for *Lactobacillus rhamnosus*	4	n.r.	16	64	1	1	8
EFSA guideline for *Bifidobacterium*	2	2	64	n.r.	1	1	8

Quantitative antibiotic sensitivity is expressed as the minimum inhibitory concentration against the microbial strains and classified as R, resistant (≥ 32 or 256 µg/ml), S, sensitive (< 0.016 µg/ml), and n.r., not required in European food safety authority (EFSA)

### Enhancement of host cell viability by cat-originated probiotics

**Fig. 1:**
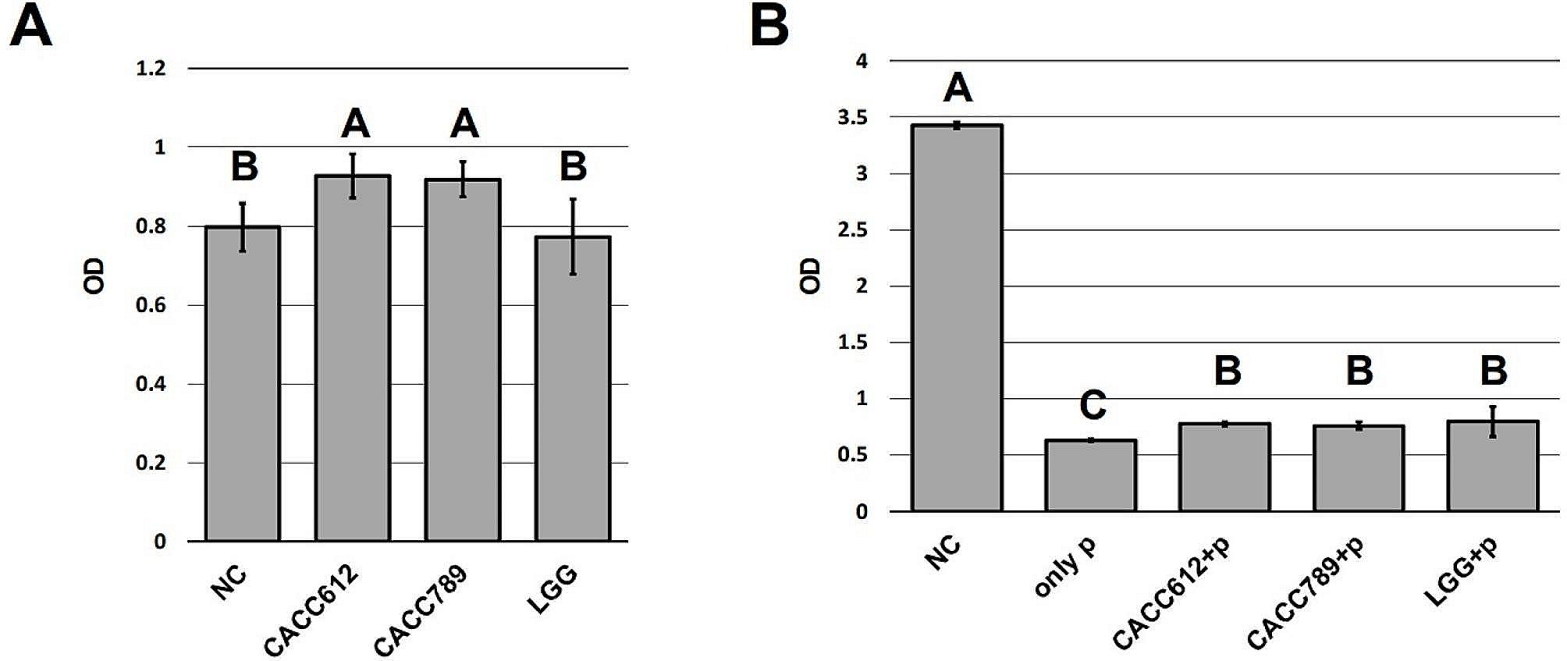
Cell viability was determined by WST-1 assay. Probiotics treatment in Fcwf-4 cells (**a**) and probiotics treatment in immune-stimulated Fcwf-4 cells (**b**). LGG (Reference strain), *Lactobacillus rhamnosus* GG (ATCC53103); NC, only bacterial broth media; p, 100 ng/ml poly(I:C) treatment

When the bacterial culture broths were co-cultured with feline macrophage cell line (Fcwf-4) to evaluate the enhancement of host cell viability by the byproducts of the probiotic bacterial strains (CACC612 and CACC789), the probiotic strains showed higher cell viability than the negative control. In addition, LGG treatment did not affect cell viability (Fig. 1a) (*P* < 0.05). These results indicated that CACC612 and CACC789 promoted feline immune cells and attenuated cell damage induced by immune stimulation.

### Attenuation of immune stimulation by cat-originated probiotics

**Fig. 2:**
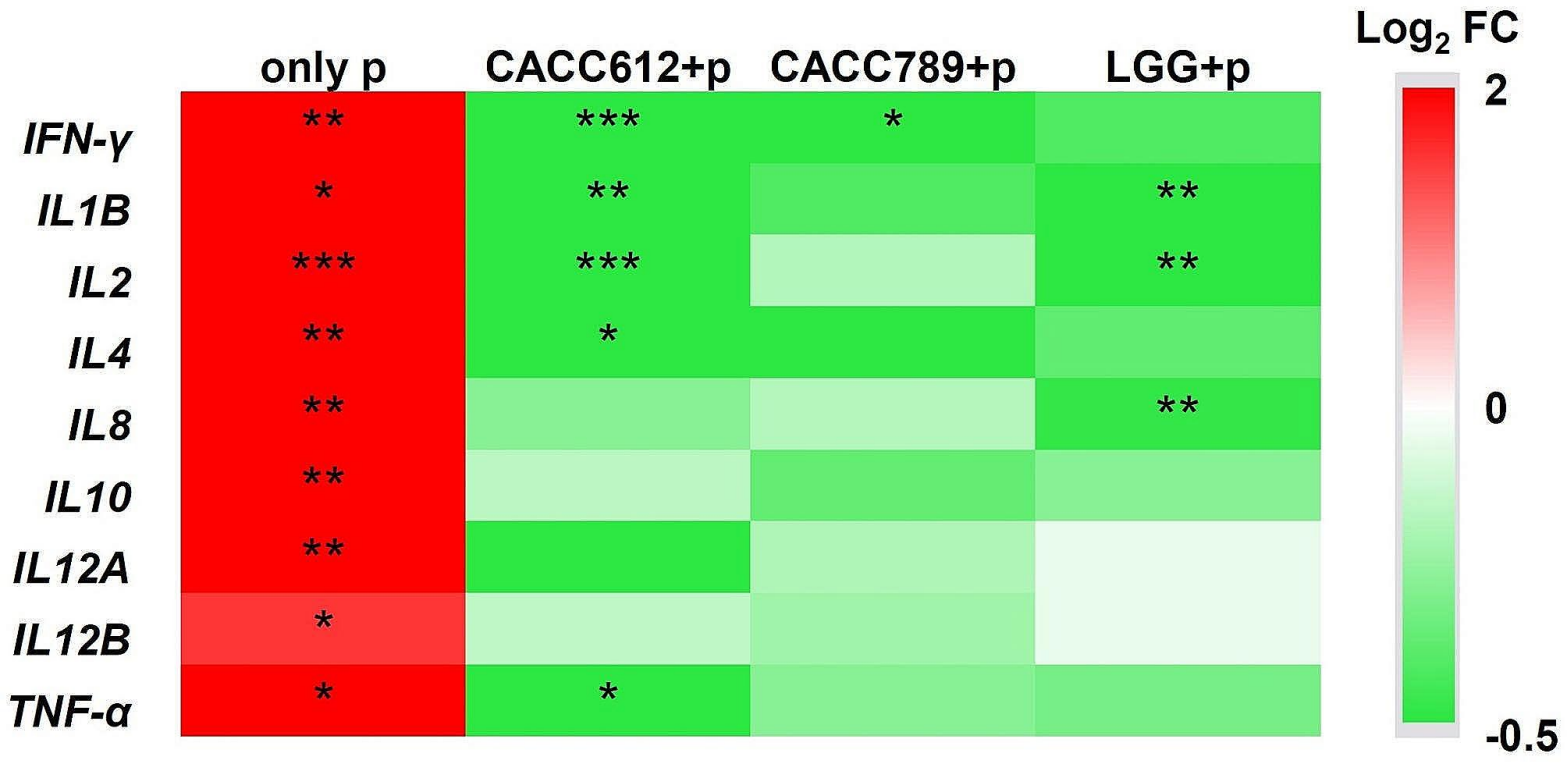
Relative expression of immune-related genes. Only Pfoldchange (FC) = Log2 (p/NT) and the other FCs = Log2 (each treatment/p). LGG (Reference strain), *Lactobacillus rhamnosus* GG (ATCC53103); NC, only bacterial broth media; p, 100 ng/ml poly(I:C) treatment; *, *P* < 0.05; **, *P* < 0.01; ***, *P* < 0.001

When immune responses were stimulated in Fcwf-4 cells using poly(I:C), cell viability was rapidly reduced; however, treatment with CACC612, CACC789, or LGG increased cell viability compared to only poly(I:C) treatment (*P* < 0.05) (Fig. 1b). Subsequently, the expression of immune-related genes, such as IFN-γ, IL1B, IL2, IL4, IL8, IL10, IL12A, IL12B, and TNF-γ, was analyzed in each treatment group; the expression of all analyzed genes was significantly increased only in the poly(I:C) treatment compared to the negative control (*P* < 0.05). In addition, treatment with CACC612, CACC789, or LGG decreased the expression of immune-related genes compared to poly(I:C) treatment alone. Notably, CACC612 significantly reduced the expression of IFN-γ, ILB1, IL2, IL4, and TNF-γ, and CACC789 decreased the expression of IFN-γ (*P* < 0.05). Additionally, LGG significantly decreased the expression of ILB1, IL2, and IL8 (*P* < 0.05) (Fig. 2). These results suggest that CACC612 and CACC789 attenuate cell damage mediated by rapid immune stimulation.

### Feeding effects of cat-originated probiotics in cats

**Fig. 3:**
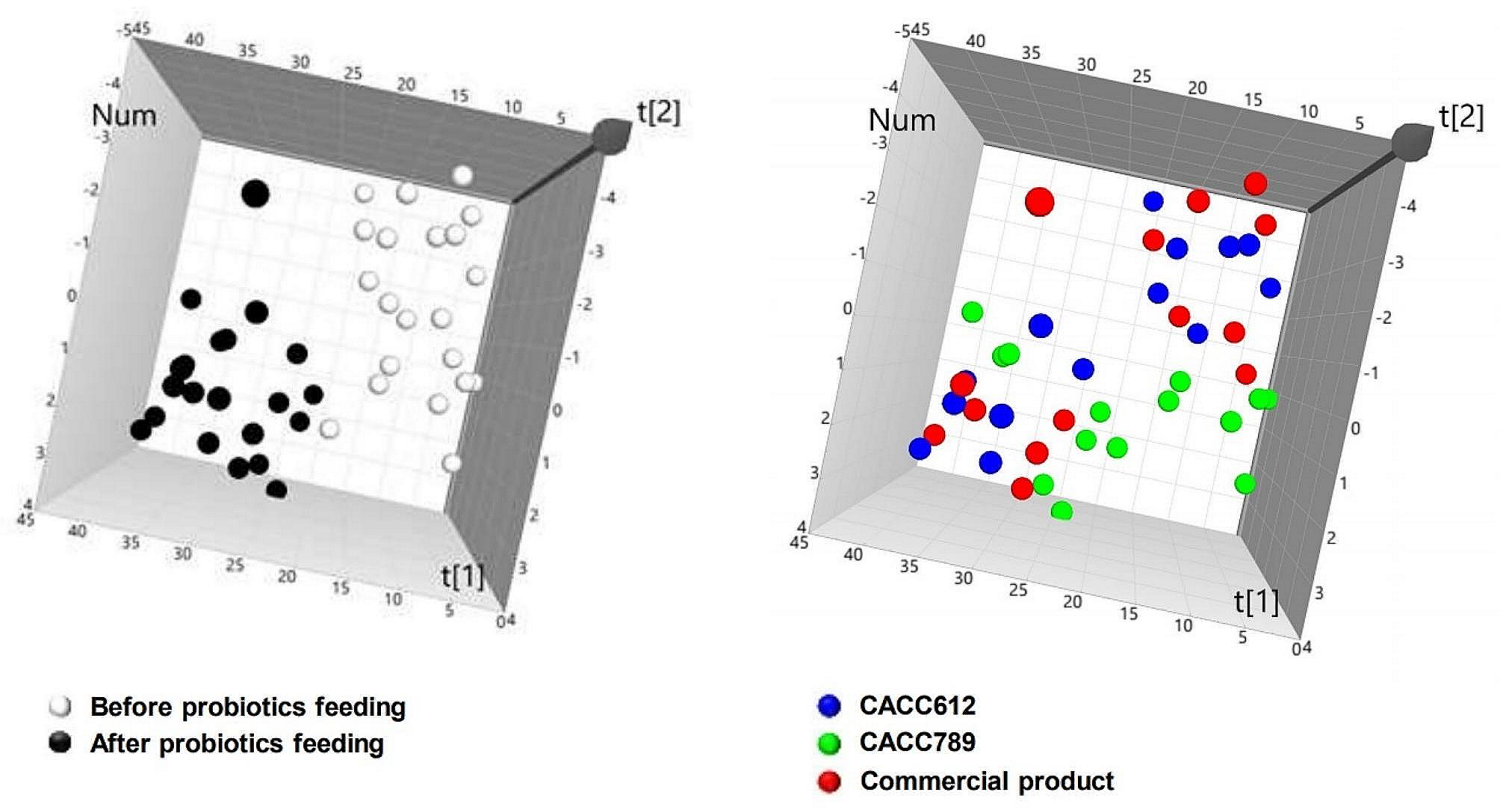
Partial Least-Squares Discriminant Analysis (PLS-DA) based on 52 parameters from analysis of whole blood and electrolyte tests before and after application of CACC612, CACC789, and the commercial product. all individuals were distributed before and after the application (left panel) and distributed before and after the application according to the application group (right panel)

To evaluate the physiological effects of probiotic bacterial strains in cats, each probiotic bacterial strain (CACC612 and CACC789) and a commercial probiotic product were fed to seven cats per experimental group for 45 days. Blood from individual cats was sampled before and after probiotic feeding. Subsequently, 52 blood parameters were examined using a complete blood count (CBC) and electrolyte tests. The examined data were collectively integrated and analyzed using principal component analysis (PLS-DA); PLS-DA results showed that individual cats were clustered before and after probiotic feeding. However, it was not separated among experimental groups (Fig. 3). These results implicated that the applied probiotics including the commercial product could contribute to the changes in blood parameters and the effects on the blood parameters might be similar among the applied probiotics including the commercial product. Sunsequently, analysis of detailed blood parameters showed that hemoglobin and mean corpuscular hemoglobin concentrations (MCHC) increased after CACC612 feeding (*P* < 0.05). Additionally, MCHC and mean platelet volume (MPV) increased after commercial product feeding; however, Mg^2+^ and nMG decreased (*P* < 0.05) (Table 6). Collectively, these results indicate that CACC612, CACC789, and the commercial probiotic product could affect the physiological status of cats and that CACC612 and the commercial product could alter blood parameters.

**Table 6:** Values of blood parameters before and after probiotics feeding

Blood parameter	CACC612							CACC789							Commercial							
	Before feeding			After feeding			P value	Before feeding			After feeding			P value	Before feeding			After feeding			P value	
Mg^2+^ (mmol/L)	0.51	±	0.03	0.46	±	0.04	0.288	0.50	±	0.03	0.53	±	0.02	0.880	**0.53**	**±**	**0.03**	**0.46**	**±**	**0.04**	**0.019**	
nMg	0.48	±	0.02	0.45	±	0.04	0.814	0.49	±	0.04	0.52	±	0.04	0.554	**0.50**	**±**	**0.04**	**0.44**	**±**	**0.03**	**0.030**	
Hemoglobin [Hb] (g/dL)	**12.23**	**±**	**1.87**	**16.64**	**±**	**4.57**	**0.018**	13.74	±	0.81	13.19	±	1.73	1.000	12.03	±	1.70	13.79	±	1.71	0.904	
MCHC (g/dL)	**29.73**	**±**	**0.75**	**31.70**	**±**	**0.53**	**0.044**	32.93	±	0.59	34.10	±	2.64	0.614	**30.16**	**±**	**0.95**	**32.24**	**±**	**0.82**	**0.026**	
MPV (fL)	10.23	±	0.31	10.50	±	0.14	0.988	10.94	±	0.80	10.86	±	0.32	1.000	**9.43**	**±**	**0.49**	**10.60**	**±**	**0.40**	**0.001**	

Bold values indicate significantly different values before and after probiotics feeding (*P* < 0.5)

## Discussion

*Lactobacillus rhamnosus*, recently classified as *Lacticaseibacillus rhamnosus*, is a representative lactic acid bacterium with ideal probiotic characteristics [28, 29]. Notably, LGG isolated from fecal samples of healthy human adults is resistant to gastric acid and bile; therefore, it survives and persists within the gastrointestinal tract, adheres to the intestinal surface, and inhibits several pathogens [30–33]. In addition, the Association of American Feed Control Officials (AAFCO) and the European Food Safety Authority (EFSA) have suggested commercially available probiotic bacteria from the *Lactobacillus*, *Bifidobacterium*, *Streptococcus*, and *Enterococcus* genera [10, 34]; the qualified presumption of safety (QPS) recommended by the EFSA includes *Lactobacillus* and *Bifidobacterium* as representative probiotics because no harmful effects have been reported following the extensive record of safe use [24, 35]. In this study, *Lactobacillus rhamnosus* CACC612 (CACC612) and *Bifidobacterium animalis* CACC789 (CACC789) were isolated from feline feces. Their probiotic attributes, such as tolerance to bile and cell adhesion activity, were superior or equivalent to LGG. Therefore, CACC612 and CACC789 could be considered as potential probiotics in cats.

Numerous studies have reported that probiotics are potential immune modulators [36]. Cytokines play key roles in the regulation of the immune response. They regulate inflammatory responses to pathogens and injury by mediating intercellular signaling. Cytokines related to inflammatory responses are largely divided into proinflammatory cytokines which are involved in the up-regulation of inflammatory reactions and anti-inflammatory cytokines which control the pro-inflammatory cytokine response. In previous studies, a single application or mixture of probiotics has shown the reduction of proinflammatory cytokines including IFN-γ IL1B, IL2, IL8, and TNF-α in various animal species and cell lines after pathogen-induced infection [37–47]. In this study, CACC612 significantly reduced the expression of IFN-γ, IL1B, IL2, IL4, and TNF-α in the immune-stimulated Fcwf-4 and CACC789 only reduced IFN-γ. These results suggested that CACC612 can attenuate more variety of proinflammatory cytokines in the immune-stimulated Fcwf-4 cell line compared to CACC789. Additionally, although both CACC612 and LGG belong to *Lactobacillus rhamnosus* species, CACC612 decreased IL4, known as an anti-inflammatory cytokine with other proinflammatory cytokines while LGG showed a significant reduction of proinflammatory cytokines such as IL1B, IL2, and IL8. Recent studies have suggested that IL4 has diverse roles besides its well-known function in immune responses [48]. Therefore, our research findings indicated that *Lactobacillus rhamnosus* CACC612 was more effective in cats compared to *Bifidobacterium animalis* subsp. *lactis* CACC789. Furthermore, the probiotic effects of CACC612 differed from those exhibited by LGG, suggesting potential host- or strain-specific effects.

Host specificity has been a suggested criterion for selecting effective probiotic candidates because of the differences in physiological structure, immune systems, and microbial composition [49, 50]. However, the efficacy of commercial probiotics has been primarily studied using human-derived probiotics based on human-optimized criteria [51]. In clinical studies, LGG may not be suitable for canine application because of its temporary persistence [52]. Additionally, canine-derived probiotics inhibit the adhesion of intestinal pathogenic bacteria to canine jejunal chyme more efficiently than non-canine strains [53]. Another study reported that *Bifidobacterium* might not play an essential role in cats compared to humans [54]. In our study, *Lactobacillus rhamnosus* (CACC612) significantly improved the hemoglobin (Hb) and mean corpuscular hemoglobin concentration (MCHC) values whereas *Bifidobacterium animalis* subsp. *lactis* CACC789 did not change blood parameter values. Hb serves as a crucial respiratory transporter, conveying oxygen from the lungs to tissues and aiding in the removal of carbon dioxide in tissues. MCHC indicates the quantity of Hb present in red blood cells. Maintaining adequate levels of Hb within red blood cells can help prevent anemia [55]. Accordingly, we proposed that *Lactobacillus rhamnosus* (CACC612) is more suitable probiotics than *Bifidobacterium animalis* subsp. *lactis* CACC789 .In conclusion, we isolated feline-derived probiotics and demonstrated their desirable characteristics. This study indicates that CACC612 and CACC789 may exhibit host- or strain-specific effects in cats, contributing to understanding the effects of probiotics and the selection of optimal probiotics for cats.

## Methods

*Recruitment of Animal Subjects’*The Institutional Animal Care and Use Committee of the Institution approved all animal procedures (CIALM 2020-01). All the methods were performed per the guidelines and regulations outlined in the protocol. In addition, informed consent was obtained from the owners of all subjects involved in the study.

### Isolation of bacterial strains from feline feces

Feces were collected from Norwegian forest cats (castrated, 12 years old) and Persian cats (castrated, 10 years old) that were privately owned and had indoor access. Feces were collected from Norwegian forest cats (castrated, 12 years old) and Persian cats (castrated, 10 years old) that were privately owned and had indoor access. From the fecal samples, each colony was isolated and subjected to 16 S rRNA sequencing as previously described [56, 57]. From the 16 S rRNA sequencing data, *Lactobacillus rhamnosus* CACC612 (CACC612) and *Bifidobacterium animalis* subsp. *lactis* CACC789 (CACC789) were newly annotated and they acquired GenBank IDs in NCBI.

### Probiotics screening

To evaluate the tolerance of bacterial strains under low pH and high bile salt concentration, the stimulation of GIT was determined in the present study using a previously described procedure with modifications [58]. For assessing the tolerance of microbial strains to acidic conditions, mMRS, BL (*Bifidobacterium* spp. culture medium) broth media was adjusted to pH 2.5 (treatment) and 6.5 (control) using 1 M HCl. Next, overnight cultured isolates (approximately 1 × 10^7^ CFU/mL) were added to each pH-adjusted medium and incubated for 2 h at 37 °C (CACC612, and CACC789) without shaking, respectively. Bile tolerance of the strains was determined on the basis of growth in mMRS and BL broth media with 0.3% and 1% oxgall (Difco, United States) for 2 h, using the same incubation temperatures and conditions described earlier for acid tolerance. All experiments were carried out under anaerobic conditions. After incubation, 10 × serial dilutions of the cultures were spread on agar plates, followed by 24 h of incubation at 37 °C. The tolerance of acid and bile for the bacterial strains was evaluated by enumerating the viable colonies and the survivability was calculated; Strains were evaluated for inhibitory effects against economically important enteropathogenic microorganisms, using a previously described disk diffusion method [59] with slight modifications. The following seven enteropathogenic bacteria were used as indicators of antibacterial activity: *Escherichia coli* K99 KCTC 2617, *Salmonella* Derby NCCP 12,238, *Salmonella* Enteritidis NCCP 14,546, *Salmonella* Typhimurium NCCP 10,438, and *Clostridium difficile* JCM1296. In brief, pathogenic strains were initially grown on appropriate media: *E. coli* was grown on Luria Bertani agar (LB), *Salmonella* spp. on Salmonella and Shigella agar (SSA), and *Clostridium difficile* on EG medium (KCTC Media No. 293, https://kctc.kribb.re.kr/en/) at 37 °C for 20 h. Diffusion disks of 8 mm diameter were appropriately overlaid on the agar and 1 × 10^6^ CFU/mL of the culture suspensions were dispensed onto the disks. The plates were incubated at 30 and 37 °C for 24 h and the diameters of the inhibition zones around each disk were measured; the sensitivity of the isolated microbial strains to 7 antibiotics including ampicillin, vancomycin, gentamicin, kanamycin, erythromycin, clindamycin, and tetracycline was assessed using the E-test MIC method (E-test bio rieux BIODISK, France); and the host cell adhesion ability of the isolated microbial strains was determined using HT-29, human intestinal cell line. Above all procedures were previously described in detail [56, 57].

### Test for host cell viability

Fcwf-4 cells (CRL-2787, ATCC, feline macrophage) were cultured per well in 6-well culture plates with 2 mL of DMEM (Gibco) with 10% FBS (Hyclone), and 1% antibiotics (1 × Antibiotic-Antimycotic, Gibco) at 37 °C, with 5% CO_2_. For testing host cell viability, Fcwf-4 cells were seeded at a density of 5 × 10^3^ cells/well in separate 96 well plates with 100 µl and incubated for 42 h and the cell confluency was reached at 80%. Cell viability was determined using the WST-1 Assay Kit (Enzo, United States). The bacterial strains were cultured for 20 h at 37 °C and then adjusted the number of cells (approximately 1 × 10^8^ CFU/mL). Each bacterial culture broth was filtered using 0.2 μm sterile membrane filter ( MilliporeSigma, USA). 10 µl of the filtered broth was added to the cells and further incubated for 4 h at 37 °C with 5% CO_2_. After that, the cells were incubated with 10 µl WST-1 reagent for 3 h. Absorbance was measured at both 450 and 650 nm (as a reference) using a UV-spectrophotometer (Tecan, Swiss) according to the manufacturer’s instructions.

### Polyinosinic: polycytidylic acid (poly(I:C)) treatment

Fcwf-4 cells were prepared at 70–80% confluency per well in 6-well culture plates for each experimental group before poly(I:C) treatment exposure. To induce immune responses, poly(I:C) (Poly(IC) HMW, InvivoGen, USA) was transfected into the prepared cells at a concentration of 0.1 µg/mL using lipofectamine (Lipofectamine 3000 Transfection Reagent, Invitrogen, Thermo Fisher Scientific). Subsequently, 200 µl (1/10 volume of culture media) of each filtered bacterial culture broth was added per well and incubated for 24 h. Next, each well was substituted with 1 ml of fresh culture media, and cell viability for each well was obtained using 100 µl WST-1 treatment.

### Expression analysis of immune-related genes in Fcwf-4 cells

RNAs were isolated from the Fcwf-4 cells in each experimental group using an RNA extraction kit (Invitrogen). For quantitative reverse transcription-polymerase chain reaction (qRT-PCR), 1 µg of total RNA was used for cDNA synthesis with Rever Tra Ace-α- first strand cDNA Synthesis Kit (Toyobo, Osaka, Japan). Sequence-specific primers (Table 1) were designed using Primer-BLAST (https://www.ncbi.nlm.nih.gov/tools/primer-blast/index.cgi?LINK_LOC=BlastHome). qRT-PCR was performed using an iCycler Real-Time PCR Detection System (Bio-Rad, Hercules, CA, USA) and SYBR Green (Bio-Rad). Non-template wells without cDNA were used as negative controls. Each sample was tested in triplicates. The PCR conditions were 95 ℃ for 3 min, followed by 40 cycles at 95 ℃ for 10 s and 60 ℃ for 30 s, using a melting curve program (increasing temperature from 65 ℃ to 95 ℃ at a rate of 0.5 ℃ per 5 s) and continuous fluorescence measurement. The qRT-PCR data were normalized relative to the expression of GAPDH and calculated using the 2 ΔΔCt method, where ΔΔCt = (Ct of the target gene – Ct of GAPDH)_treatment_ – (Ct of the target gene – Ct of GAPDH)_control_ [60].

### Clinical trial

When a total of 21 cats that were privately owned and had indoor access were recruited for the clinical trial, they ranged from kittens at 6 months old to adult cats at 6 years old and had a ratio of male to female, 1 : 1.1. Subsequently, they were randomly and evenly grouped into three experimental groups (CACC612-feeding, CACC789-feeding, and commercial product-feeding). Clinical data were collected and analyzed at the MAY Animal Medical Center, Jeonju, Korea. The commercial product (Real bifidus cat™, Estien Corp, South Korea) was chosen among probiotic products for cats; CACC612 and CACC789 were cultured in mMRS broth (DifcoTM Lactobacilli MRS broth, BD Company, USA) and BL broth (*Bifidobacterium* Selective broth, MB cell, South Korea) under anaerobic conditions (5% hydrogen, 5% carbon dioxide, and 90% nitrogen) at 37 °C for 48 h, respectively and then lyophilized. The probiotic products consisted of 5% fructooligosaccharide, 10% skimmed milk, 15% trehalose, 0.5% glycerin, 1% NaCl, and one of the following bacterial strains: CACC612 and CACC789. Each experimental group was administered 0.2 g of probiotic product, including 10^8^ bacteria, daily for 45 days. The powdered probiotic product (0.2 g) was individually sealed in plastic medicine bags. The powder was dissolved in 1 ml water and fed into a 1 ml syringe. No significant adverse symptoms were reported during clinical trials. Serum samples were collected from cats before feeding the probiotic products and 45 days after feeding with the probiotic products. Serum samples were analyzed using a complete blood count (CBC) and electrolyte tests according to standard protocols.

### Statistical analysis

Statistical evaluation of the data was performed using analysis of variance (ANOVA) with a general linear model for a randomized complete block design. All treatments were performed in triplicate, and Tukey’s HSD test was used to define the mean differences between specific treatments. The statistical significance (*P* < 0.05, *P* < 0.01, or *P* < 0.001) of the differences was determined. All analyses were conducted using JMP 14.3.0 (SAS Institute Inc. software (NC, United States)).

## Data Availability

The datasets used and/or analyzed in the current study are willing to be provided by the corresponding author upon any request.

## Abbreviations

Hb: hemoglobin
IFN-γ: interferon gamma
IL1B: interleukin 1 beta
IL2: interleukin 2
IL4: interleukin 4
IL8: interleukin 8
IL10: interleukin 10
IL12A: interleukin 12 subunit alpha
IL12B: interleukin 12 subunit alpha
TNF-α: tumour necrosis factor alpha
LGG: *Lactobacillus rhamnosus* GG
MCHC: mean corpuscular hemoglobin concentration
MIC: minimum inhibitory concentration
MPV: mean platelet volume
PLS-DA: partial least-squares discriminant analysis
poly(I: C):polyinosinic-polycytidylic acid

## References

[1] FAO/WHO. Evaluation of health and nutritional properties of powder milk and live lactic acid bacteria. : FAO Rome; 2001: 1–34.

[2] Sarao LK, Arora M (2017). Probiotics, prebiotics, and microencapsulation: a review. Crit Rev Food Sci Nutr.

[3] Wieers G, Belkhir L, Enaud R, Leclercq S, Philippart de Foy JM, Dequenne I, de Timary P, Cani PD (2019). How Probiotics affect the Microbiota. Front Cell Infect Microbiol.

[4] Hill C, Guarner F, Reid G, Gibson GR, Merenstein DJ, Pot B, Morelli L, Canani RB, Flint HJ, Salminen S (2014). Expert consensus document. The International Scientific Association for Probiotics and Prebiotics consensus statement on the scope and appropriate use of the term probiotic. Nat Rev Gastroenterol Hepatol.

[5] O’Connor PM, Kuniyoshi TM, Oliveira RP, Hill C, Ross RP, Cotter PD (2020). Antimicrobials for food and feed; a bacteriocin perspective. Curr Opin Biotechnol.

[6] Fiore W, Arioli S, Guglielmetti S. The neglected Microbial Components of Commercial Probiotic formulations. Microorganisms 2020, 8(8). 10.3390/microorganisms8081177 PMC7464440 32756409

[7] Acuff H, Aldrich CG. A Review of Application Strategies and Efficacy of Probiotics in Pet Food. *Antibiotics and Probiotics in Animal Food-Impact and Regulation* 2022.

[8] Song D, Ibrahim S, Hayek S (2012). Recent application of probiotics in food and agricultural science. Probiotics.

[9] Holzapfel WH, Haberer P, Geisen R, Björkroth J, Schillinger U (2001). Taxonomy and important features of probiotic microorganisms in food and nutrition. Am J Clin Nutr.

[10] AAFCO. AAFCO official publication. In.: Association of American Feed Control Officials Champaign (IL); 2015.

[11] Mattia A, Merker R (2008). Regulation of probiotic substances as ingredients in foods: premarket approval or generally recognized as safe notification. Clin Infect Dis.

[12] Salminen S, von Wright A, Morelli L, Marteau P, Brassart D, de Vos WM, Fonden R, Saxelin M, Collins K, Mogensen G (1998). Demonstration of safety of probiotics -- a review. Int J Food Microbiol.

[13] Grzeskowiak L, Collado MC, Beasley S, Salminen S (2014). Pathogen exclusion properties of canine probiotics are influenced by the growth media and physical treatments simulating industrial processes. J Appl Microbiol.

[14] Biagi G, Cipollini I, Bonaldo A, Grandi M, Pompei A, Stefanelli C, Zaghini G (2013). Effect of feeding a selected combination of galacto-oligosaccharides and a strain of Bifidobacterium pseudocatenulatum on the intestinal microbiota of cats. Am J Vet Res.

[15] Gonzalez-Ortiz G, Castillejos L, Mallo JJ, Angels Calvo-Torras M, Dolores Baucells M (2013). Effects of dietary supplementation of Bacillus amyloliquefaciens CECT 5940 and Enterococcus faecium CECT 4515 in adult healthy dogs. Arch Anim Nutr.

[16] Kumar S, Pattanaik AK, Sharma S, Jadhav SE, Dutta N, Kumar A (2017). Probiotic potential of a Lactobacillus bacterium of Canine Faecal-Origin and its impact on Select Gut Health indices and Immune Response of Dogs. Probiotics Antimicrob Proteins.

[17] Bybee SN, Scorza AV, Lappin MR (2011). Effect of the probiotic Enterococcus faecium SF68 on presence of diarrhea in cats and dogs housed in an animal shelter. J Vet Intern Med.

[18] Marsella R, Santoro D, Ahrens K (2012). Early exposure to probiotics in a canine model of atopic dermatitis has long-term clinical and immunological effects. Vet Immunol Immunopathol.

[19] Ohshima-Terada Y, Higuchi Y, Kumagai T, Hagihara A, Nagata M (2015). Complementary effect of oral administration of Lactobacillus paracasei K71 on canine atopic dermatitis. Vet Dermatol.

[20] Strompfova V, Laukova A, Gancarcikova S (2012). Effectivity of freeze-dried form of Lactobacillus fermentum AD1-CCM7421 in dogs. Folia Microbiol (Praha).

[21] Baffoni L. Probiotics and prebiotics for the health of companion animals. Probiotics Prebiotics Anim Health food Saf 2018:175–95.

[22] Jugan MC, Rudinsky AJ, Parker VJ, Gilor C (2017). Use of probiotics in small animal veterinary medicine. J Am Vet Med Assoc.

[23] McCoy S, Gilliland SE (2007). Isolation and characterization of Lactobacillus species having potential for use as probiotic cultures for dogs. J Food Sci.

[24] Lee D, Goh TW, Kang MG, Choi HJ, Yeo SY, Yang J, Huh CS, Kim YY, Kim Y (2022). Perspectives and advances in probiotics and the gut microbiome in companion animals. J Anim Sci Technol.

[25] Rudenko P, Vatnikov Y, Sachivkina N, Rudenko A, Kulikov E, Lutsay V, Notina E, Bykova I, Petrov A, Drukovskiy S et al. Search for promising strains of probiotic microbiota isolated from different biotopes of healthy cats for Use in the control of Surgical infections. Pathogens 2021, 10(6). 10.3390/pathogens10060667 PMC8228694 34071725

[26] Abdou AM, Hedia RH, Omara ST, Mahmoud MAE, Kandil MM, Bakry MA (2018). Interspecies comparison of probiotics isolated from different animals. Vet World.

[27] Panel EF. Guidance on the assessment of bacterial susceptibility to antimicrobials of human and veterinary importance. EFSA Journal 2012; 10 (6): 2740, 10 pp. In.; 2012.

[28] Zheng J, Wittouck S, Salvetti E, Franz C, Harris HMB, Mattarelli P, O’Toole PW, Pot B, Vandamme P, Walter J (2020). A taxonomic note on the genus Lactobacillus: description of 23 novel genera, emended description of the genus Lactobacillus Beijerinck 1901, and union of Lactobacillaceae and Leuconostocaceae. Int J Syst Evol Microbiol.

[29] Mathipa-Mdakane MG, Thantsha MS. Lacticaseibacillus rhamnosus: a suitable candidate for the construction of Novel Bioengineered probiotic strains for targeted Pathogen Control. Foods 2022, 11(6). 10.3390/foods11060785 PMC8947445 35327208

[30] de Champs C, Maroncle N, Balestrino D, Rich C, Forestier C (2003). Persistence of colonization of intestinal mucosa by a probiotic strain, Lactobacillus casei subsp. rhamnosus Lcr35, after oral consumption. J Clin Microbiol.

[31] Forestier C, De Champs C, Vatoux C, Joly B (2001). Probiotic activities of Lactobacillus casei rhamnosus: in vitro adherence to intestinal cells and antimicrobial properties. Res Microbiol.

[32] Doron S, Snydman DR, Gorbach SL (2005). Lactobacillus GG: bacteriology and clinical applications. Gastroenterol Clin North Am.

[33] Papizadeh M, Nahrevanian H, Rohani M, Hosseini SN, Shojaosadati SA (2016). Lactobacillus rhamnosus Gorbach-Goldin (GG): a top well-researched probiotic strain. J Med Bacteriol.

[34] EFSA (2005). Opinion of the Scientific Committee on a request from EFSA related to a generic approach to the safety assessment by EFSA of microorganisms used in food/feed and the production of food/feed additives. EFSA J.

[35] Hempel S, Newberry S, Ruelaz A, Wang Z, Miles JN, Suttorp MJ, Johnsen B, Shanman R, Slusser W, Fu N (2011). Safety of probiotics used to reduce risk and prevent or treat disease. Evid Rep Technol Assess (Full Rep).

[36] Azad MAK, Sarker M, Wan D. Immunomodulatory Effects of Probiotics on Cytokine Profiles. *Biomed Res Int* 2018, 2018:8063647. 10.1155/2018/8063647 PMC6218795 30426014

[37] Chen Q, Tong C, Ma S, Zhou L, Zhao L, Zhao X (2017). Involvement of MicroRNAs in Probiotics-Induced reduction of the Cecal inflammation by Salmonella Typhimurium. Front Immunol.

[38] Garcia-Castillo V, Zelaya H, Ilabaca A, Espinoza-Monje M, Komatsu R, Albarracin L, Kitazawa H, Garcia-Cancino A, Villena J (2018). Lactobacillus fermentum UCO-979 C beneficially modulates the innate immune response triggered by Helicobacter pylori infection in vitro. Benef Microbes.

[39] Wang S, Peng Q, Jia HM, Zeng XF, Zhu JL, Hou CL, Liu XT, Yang FJ, Qiao SY (2017). Prevention of Escherichia coli infection in broiler chickens with Lactobacillus plantarum B1. Poult Sci.

[40] Zhang F, Zeng X, Yang F, Huang Z, Liu H, Ma X, Qiao S (2013). Dietary N-Carbamylglutamate supplementation boosts intestinal mucosal immunity in Escherichia coli Challenged piglets. PLoS ONE.

[41] Yu HJ, Liu W, Chang Z, Shen H, He LJ, Wang SS, Liu L, Jiang YY, Xu GT, An MM (2015). Probiotic BIFICO cocktail ameliorates Helicobacter pylori induced gastritis. World J Gastroenterol.

[42] Chen YH, Tsai WH, Wu HY, Chen CY, Yeh WL, Chen YH, Hsu HY, Chen WW, Chen YW, Chang WW et al. Probiotic Lactobacillus spp. act against Helicobacter pylori-induced inflammation. J Clin Med 2019, 8(1). 10.3390/jcm8010090 PMC6352136 30646625

[43] Boltin D (2016). Probiotics in Helicobacter pylori-induced peptic ulcer disease. Best Pract Res Clin Gastroenterol.

[44] Song H, Zhou L, Liu D, Ge L, Li Y (2019). Probiotic effect on Helicobacter pylori attachment and inhibition of inflammation in human gastric epithelial cells. Exp Ther Med.

[45] Souza RFS, Rault L, Seyffert N, Azevedo V, Le Loir Y, Even S (2018). Lactobacillus casei BL23 modulates the innate immune response in Staphylococcus aureus-stimulated bovine mammary epithelial cells. Benef Microbes.

[46] Martins FS, Elian SD, Vieira AT, Tiago FC, Martins AK, Silva FC, Souza EL, Sousa LP, Araujo HR, Pimenta PF (2011). Oral treatment with Saccharomyces cerevisiae strain UFMG 905 modulates immune responses and interferes with signal pathways involved in the activation of inflammation in a murine model of typhoid fever. Int J Med Microbiol.

[47] Martins FS, Rodrigues ACP, Tiago FCP, Penna FJ, Rosa CA, Arantes RME, Nardi RMD, Neves MJ, Nicoli JR (2007). Saccharomyces cerevisiae strain 905 reduces the translocation of Salmonella enterica serotype typhimurium and stimulates the immune system in gnotobiotic and conventional mice. J Med Microbiol.

[48] Luzina IG, Keegan AD, Heller NM, Rook GA, Shea-Donohue T, Atamas SP (2012). Regulation of inflammation by interleukin-4: a review of alternatives. J Leukoc Biol.

[49] Dogi CA, Perdigon G (2006). Importance of the host specificity in the selection of probiotic bacteria. J Dairy Res.

[50] Park H, Yeo S, Arellano K, Kim HR, Holzapfel W. Role of the gut microbiota in health and disease. Probiotics Prebiotics Anim Health food Saf 2018:35–62.

[51] Yeo S, Lee S, Park H, Shin H, Holzapfel W, Huh CS (2016). Development of putative probiotics as feed additives: validation in a porcine-specific gastrointestinal tract model. Appl Microbiol Biotechnol.

[52] Weese JS, Anderson ME (2002). Preliminary evaluation of Lactobacillus rhamnosus strain GG, a potential probiotic in dogs. Can Vet J.

[53] Rinkinen M, Jalava K, Westermarck E, Salminen S, Ouwehand AC (2003). Interaction between probiotic lactic acid bacteria and canine enteric pathogens: a risk factor for intestinal Enterococcus faecium colonization?. Vet Microbiol.

[54] Masuoka H, Shimada K, Kiyosue-Yasuda T, Kiyosue M, Oishi Y, Kimura S, Ohashi Y, Fujisawa T, Hotta K, Yamada A (2017). Transition of the intestinal microbiota of cats with age. PLoS ONE.

[55] Marengo-Rowe AJ (2006). Structure-function relations of human hemoglobins. Proc (Bayl Univ Med Cent).

[56] Kim JA, Bayo J, Cha J, Choi YJ, Jung MY, Kim DH, Kim Y (2019). Investigating the probiotic characteristics of four microbial strains with potential application in feed industry. PLoS ONE.

[57] Jang HJ, Son S, Kim JA, Jung MY, Choi YJ, Kim DH, Lee HK, Shin D, Kim Y (2021). Characterization and functional test of Canine Probiotics. Front Microbiol.

[58] Liong MT, Shah NP (2005). Acid and bile tolerance and cholesterol removal ability of lactobacilli strains. J Dairy Sci.

[59] Tagg JR, McGiven AR (1971). Assay system for bacteriocins. Appl Microbiol.

[60] Livak KJ, Schmittgen TD (2001). Analysis of relative gene expression data using real-time quantitative PCR and the 2(-Delta Delta C(T)) method. Methods.

[61] Srivastava M, Ranjan A, Choudhary JK, Tripathi MK, Verma S, Dixit VK, Nath G, Jain AK (2014). Role of proinflammatory cytokines (interferon gamma) and anti-inflammatory cytokine (interleukin-10) gene polymorphisms in chronic hepatitis B infection: an Indian scenario. J Interferon Cytokine Res.

[62] Zhang JM, An J (2007). Cytokines, inflammation, and pain. Int Anesthesiol Clin.

[63] Ng PC, Li K, Wong RP, Chui K, Wong E, Li G, Fok TF (2003). Proinflammatory and anti-inflammatory cytokine responses in preterm infants with systemic infections. Arch Dis Child Fetal Neonatal Ed.

[64] Sikora JP, Chlebna-Sokol D, Krzyzanska-Oberbek A (2001). Proinflammatory cytokines (IL-6, IL-8), cytokine inhibitors (IL-6sR, sTNFRII) and anti-inflammatory cytokines (IL-10, IL-13) in the pathogenesis of sepsis in newborns and infants. Arch Immunol Ther Exp (Warsz).

[65] Trinchieri G (1995). Interleukin-12: a proinflammatory cytokine with immunoregulatory functions that bridge innate resistance and antigen-specific adaptive immunity. Annu Rev Immunol.

